# Moderate doses of conjugated linoleic acid reduce fat gain, maintain insulin sensitivity without impairing inflammatory adipose tissue status in mice fed a high-fat diet

**DOI:** 10.1186/1743-7075-7-5

**Published:** 2010-01-20

**Authors:** Pilar Parra, Andreu Palou, Francisca Serra

**Affiliations:** 1 University of the Balearic Islands, Cra Valldemossa Km 7,5, E-07122, Palma de Mallorca, Spain

## Abstract

**Background:**

The enrichment of diet with nutrients with potential benefits on body composition is a strategy to combat obesity. Conjugated linoleic acid (CLA) due its beneficial effects on body composition and inflammatory processes becomes an interesting candidate, since the promotion and impairment of obesity is closely linked to a low-grade inflammation state of adipose tissue. Previously we reported the favourable effects of moderate doses of CLA mixture on body composition and inflammatory status of adipose tissue in mice fed a standard-fat diet. In the present study we assessed the potential beneficial effects of CLA mixture (*cis*-9, *trans*-11 and *trans*-10, *cis*-12, 50:50) in mice fed a high-fat diet.

**Methods:**

Two doses were assayed: 0.15 g (CLA1) and 0.5 g CLA/kg body weight (CLA2) for the first 30 days of the study and then animals received a double amount for another 35 days.

**Results:**

The lowest dose (CLA1) had minor effects on body composition, plasma parameters and gene expression. However, a clear reduction in fat accumulation was achieved by CLA2, accompanied by a reduction in leptin, adiponectin and non-esterified fatty acids (NEFA) plasma concentrations. Insulin sensitivity was maintained despite a slight increase in fasting glucose and insulin plasma concentrations. The study of gene expression both in adipocytes and in the stromal vascular fraction (SVF) suggested that CLA may reduce either the infiltration of macrophages in adipose tissue or the induction of expression of pro-inflammatory cytokines.

**Conclusion:**

In conclusion, the use of moderate doses of an equimolar mix of the two main CLA isomers reduces body fat content, improves plasma lipid profile, maintains insulin sensitivity (despite a moderate degree of hyperinsulinaemia) without the promotion of inflammatory markers in adipose tissue of mice fed a high-fat diet.

## Background

CLA refers to a group of positional and geometric isomers of linoleic acid and has been extensively studied due to its potential beneficial effects in several diseases including cancer, atherosclerosis, diabetes and obesity [1,2]. It has been suggested that the anti-carcinogenic and anti-atherosclerosis effect of CLA stems from its anti-inflammatory properties. One of the mechanisms proposed by which CLA could exert its anti-inflammatory effect is by the negative regulation of gene expression of inflammation mediators [3]. The increased size of adipose depots in obesity is related to a certain degree of inflammation which may be involved in the pathophysiology of obesity-associated disorders. This low-grade inflammatory state has been associated with the progressive infiltration of macrophages into adipose tissue, which may be the main source of pro-inflammatory cytokines and associated insulin resistance in obesity [4,5]. Furthermore, most studies conducted in animals demonstrated that CLA reduces body fat mass [6] with the *trans*-10, *cis*-12 CLA isomer mainly responsible for this effect [1,7]. Therefore CLA, due to its beneficial potential effects on both body composition and inflammation, becomes an interesting nutritional strategy in the treatment of obesity. However, in some studies conducted in mice —the most sensitive species— fat loss triggered by CLA was accompanied by deleterious side effects such as insulin resistance, hyperinsulinaemia and liver steatosis [8-11]. Both beneficial and detrimental effects of CLA supplementation are more modest or less evident in human studies. However, a recent meta-analysis of human studies supports a modest effect of CLA reducing body fat [12].

Controversial results about the anti-inflammatory properties of CLA also exist. *In vitro *data demonstrate that *trans*-10, *cis*-12 CLA activates NFκB- and ERK1/2-dependent IL-6, IL-8, and TNFα production, which impairs adipogenic gene expression and glucose uptake [13]. Furthermore, supplementation with *trans*-10, *cis*-12 CLA promotes macrophage infiltration into adipose tissue, contributing to adipose tissue inflammation and insulin resistance [14]. In contrast, treatment with *cis*-9, *trans*-11 CLA reduces macrophage infiltration and attenuates the inflammatory profile of obese adipose tissue [15]. Interestingly, we have shown that the use of moderate doses of an approximately equimolar mixture of both main CLA isomers achieves a modest reduction of fat gain, ameliorates macrophage infiltration into adipose tissue and expression of pro-inflammatory cytokines, therefore, contributing to preserve adipose function [16].

In the present study, we analyze the potential effects of moderate doses of CLA mixture on body composition and insulin sensitivity, as well as on adipose tissue inflammatory profile in mice fed a high-fat diet.

## Methods

### Animals

Male mice (C57BL/6J) from Charles River (Barcelona, Spain) were housed in groups of four in plastic cages, acclimated to 22°C with a 12 h light/12 h dark cycle. Animals were fed *ad libitum *with a high-fat diet (D12451, Research Diets Inc, New Brunswick) which contains 45% calorie content as fat, 35% calorie content as carbohydrate and the remaining 20% as protein. Food intake and body weight were recorded every three days during the experiment. Total calories consumed was measured for each cage and expressed as the average of the two cages per group. Fresh food was provided to the mice biweekly. At 30 days of treatment, animals were starved for 3 h, tail blood samples were obtained to perform plasma determinations and were then submitted to the insulin tolerance test (ITT). 35 days later, animals were sacrificed under fasting conditions (10 h).

All experimental procedures were performed according to both national and institutional guidelines for animal care and use.

### CLA Treatment

The CLA used was Tonalin^® ^TG 80 derived from safflower oil (kindly provided by Cognis). Tonalin is composed of triglycerides containing approximately 80% CLA with a 50:50 ratio of the active CLA isomers *cis*-9, *trans*-11 and *trans*-10, *cis*-12.

Mice weighing 20 ± 0.2 g (5-week-old) were randomly assigned to three experimental oral treatments: sunflower oil (control group, n = 8), CLA1 (n = 8) or CLA2 (n = 8) for 65 days. For the first 30 days, two doses of CLA were assessed: CLA1 (0.15 g CLA/kg body weight) and CLA2 (0.50 g CLA/kg body weight), taking the weight of the animals at the beginning of the experiment as a reference. After 30 days of treatment and until the end of the experiment, the corresponding dose of each group was doubled. Therefore, animals received a daily amount of Tonalin equivalent to 3 mg CLA/animal in CLA1 group and 10 mg/animal in CLA2 group for the first 30 days and 6 mg CLA/animal in CLA1 group and 20 mg/animal in CLA2 group for the last 35 days of treatment. An adequate amount of commercial sunflower oil was given to the animals to achieve isocaloric load between groups.

### Insulin tolerance test

ITT was performed on day 30 of the study after 3 h fast. Recombinant human insulin (Humulin R; Eli Lilly, Spain), previously diluted in 0.9% saline, was intraperitoneally injected (0.8 U/kg body weight). Blood glucose concentration was determined from tail blood samples before and at 15, 30, 60, 90, and 120 min postinjection using an Accu Check Sensor (Roche Diagnostics, Barcelona, Spain). The area under the curve for each mice was calculated using the KaleidaGraph software version 3.0 (Synergy Software, Reading, PA, U.S.A.), and the mean value ± SEM calculated for each group.

### Sacrifice and tissue sampling

Mice were anaesthetised by intraperitoneal injection of a mixture of xilacine (10 mg/kg body weight) and ketamine (100 mg/kg body weight) and blood was collected by cardiac puncture. Liver, brown and white adipose depots were rapidly removed, weighed, rinsed with saline containing 0.1% diethyl pyrocarbonate (Sigma, Madrid, Spain), frozen with nitrogen liquid and stored at -70°C. Blood collected by cardiac puncture with heparinized syringe and needle (0.2% heparin diluted with saline, Sigma, Madrid, Spain) was centrifuged at 1000 g for 10 min at 4°C and plasma obtained was stored at -70°C for later analysis.

### Plasma analysis

Adiponectin and insulin plasma concentrations were measured using a rat/mouse adiponectin ELISA kit (Phoenix Europe GmbH, Karlsruhe, Germany) and Insulin Mouse Ultrasensitive ELISA kit (DRGInstruments GmbH, Marburg, Germany) respectively. Resistin and leptin plasma concentrations were also assessed by ELISA using the following commercial kits: Mouse Resistin Quantikine ELISA kit and Mouse Leptin Quantikine ELISA kit (R&D Systems, Minneapolis, MN, USA). Commercial enzymatic colorimetric kits were used for the determination of plasma NEFA (Wako Chemicals GmbH, Neuss, Germany) and circulating concentrations of triglycerides (Sigma Diagnostics, Madrid, Spain).

### Hepatic triglyceride quantification

A sample of liver (200-300 mg) was homogenized in PBS (1:2, wt:v) using a polytron homogenizer. Homogenates were centrifuged at 500 *g *for 10 min and the supernatant was used for the quantification. Total triglyceride levels were measured using a commercial enzymatic colorimetric kit following standard procedures (Sigma Diagnostics, Madrid, Spain).

### Isolation of mature adipocytes and SVF from epididymal fat depots

Fresh epididymal white adipose tissue was digested with collagenase and after filtration and washing steps the SVF and the mature adipocyte-enriched fraction were obtained following the protocol previously described [16].

### RT-PCR reaction analysis

Total RNA from mature adipocytes and SVF were extracted using the RNAeasy Mini Kit from Qiagen (Barcelona, Spain). RNA was quantified using the NanoDrop^®^Spetrophotometer ND-1000. RT-PCR was used to measure mRNA expression levels of target genes. Aliquots of 0.5 μg of total RNA (in a final volume of 10 μL) were denatured at 90°C for 1 min and then reverse-transcribed to cDNA using MuLV reverse transcriptase (Applied Biosystem, Madrid, Spain) at 42°C for 60 min, with a final step of 5 min at 99°C in a Perkin-Elmer 9700 Thermal Cycler (PerkinElmer, Wellesley, MA). RT-PCR was completed using the LightCycler System with SYBR Green I (Roche Diagnostic Gmbh, Mannheim, Germany). Primer sequences are listed in Table 1. All primers were purchased from Sigma (Madrid, Spain). Each PCR was performed in a total volume of 8 μL, made from diluted cDNA template, forward and reverse primers (1 μmol/L each), and SYBR Green I master mix (including Taq polymerase, reaction buffer, MgCl_2_, SYBR Green I dye, and dNTP mix). In order to verify the purity of the products, a melting curve was produced after each run by increasing the temperature of the reaction mixtures up to 95°C, by 0.1°C/s, starting at 55°C for 10 s. PCR products were also analyzed by electrophoresis in an ethidium bromide-stained agarose gel to check that a single amplicon of the expected size was indeed obtained.

**Table 1:** Gene-specific primer sequences used in real-time PCR amplification

Gene	Primer sequence (5' → 3')	Product length (bp)	Primer efficiency
*Adiponectin*	F: GCTCAGGATGCTACTGTTG	255	1.9
	R: TCTCACCCTTAGGACCAAG		

*Leptin*	F: TTGTCACCAGGATCAATGACATTT	106	1.9
	R: GACAAACTCAGAATGGGGTGAAG		

*MCP1*	F: GCTCTCTCTTCCTCCACCAC	208	1.8
	R: GCTTCTTTGGGACACCTGCT		

*Emr1*	F: TTTCCTCGCCTGCTTCTTC	222	1.8
	R: CCCCGTCTCTGTATTCAACC		

*IL-6*	F: TGGGAAATCGTGGAAATGAG	249	1.9
	R: GAAGGACTCTGGCTTTGTCTT		

*TNFα*	F: CGTCGTAGCAAACCACCAA	145	1.7
	R: GAGAACCTGGGAGTAGACAAGG		

*iNOS*	F: GGCAGCTACTGGGTCAAAGA	172	1.8
	R: TCTGAGGGCTGACACAAGG		

*18S*	F: CGCGGTTCTATTTTGTTGGT	219	1.9
	R: AGTCGGCATCGTTTATGGTC		

F, forward; R, reverse. Target genes: adiponectin; leptin; monocyte chemotactic protein-1 (*MCP1*); epidermal growth factor module-containing mucin-like receptor 1 (*Emr1*); interleukin-6 (*IL-6*); tumor necrosis factor alpha (*TNFα*); inducible nitric oxide synthase (*iNOS*). *18S *rRNA was used for normalization.

The relative quantification of each target gene (*adiponectin*, *leptin*, *MPC1*, *Emr1*, *IL-6*, *TNFα*, and *iNOS*) was calculated based on efficiency and the crossing point deviation of an unknown sample versus a control, and normalized by the expression of the reference housekeeping gene *18S *rRNA [17]. Results from CLA treated groups were expressed as fold induction relative to the control group. Data were expressed using both mRNA concentration in each cellular fraction and total mRNA content.

### Statistical analysis

Data are presented as means ± SEM. Repeated-measures ANOVA was used to determine differences in body weight gain. One-way ANOVA was used to determine the significance of the differences in tissue weights, plasma concentrations of metabolites, mRNA abundance and levels with different treatments. If there was a significant difference, a Least Significant Difference (LSD) test was used to determine the particular effect that caused that difference. *P *< 0.05 was statistically significant, and different superscripts discriminate differences between groups. The analysis was performed using the SPSS program for Windows version 14 (SPSS, Chicago, IL, USA).

## Results

### Body and tissue weights and energy intake

**Figure 1:**
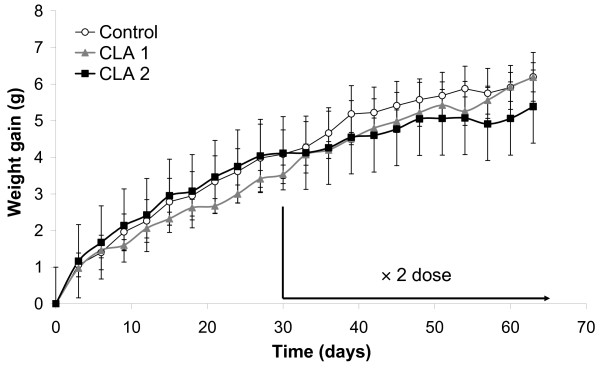
**Effects of CLA on body weight gain in mice**. Mice received a daily dose of CLA equivalent to 3 mg CLA/animal in CLA1 group and 10 mg/animal in CLA2 group for the first 30 d and 6 mg CLA/animal in CLA1 group and 20 mg/animal in CLA2 group for the last 35 d of treatment. Data are means ± SEM of 8 mice. Repeated-measures analysis of variance of body weight gain associated with CLA treatment was significant with respect to the control (*P *< 0.05). No differences between doses were found. x2dose: indicates the point from which the double dose was given.

After 30 d of CLA treatment, no evident effects on the rate of body weight gain were observed (Figure 1). Since circulating leptin levels are proportional to overall adipose mass rather than body weight, plasma leptin concentration was also determined at this time-point and no differences between groups were found. As previously suggested, fat content in diet could determine the effectiveness of CLA doses [18]. Therefore, considering that the amount of CLA administered didn't seem to have a significant effect on body fat content, we decided to double the doses from day 30 onwards. Accordingly, mice started to receive 251 mg CLA/kg body weight and day in CLA1 group and 414 mg CLA/kg body weight and day in CLA2 group until the end of the experiment.

Then, body weight reached at the end of the treatment was still not statistically different between control and CLA supplemented animals. However, lower body weight gain was observed during the treatment by CLA (Figure 1) (repeated-measures ANOVA: *P *< 0.05, effect of time × treatment) and the effects were more noticeable from day 30 onwards, with doubled doses, and in the CLA2 group, in which the increase in body weight gain for the last 30 days of study was 39% lower than in control group.

In the course of the study, no differences in total energy consumed were found between groups (2428 ± 119 in control, 2263 ± 104 in CLA1 and 2382 ± 5 kJ/animal in CLA2 group, each group n = 8). Adiposity was significantly reduced with the highest dose of CLA (47% lower vs. control group, *P *< 0.001) and weights of epididymal, retroperitoneal and brown adipose tissue were significantly lower in this group (Table 2). This effect was more marked in retroperitoneal (67% lower) and epididymal (56% lower) depots than in brown adipose tissue (20% lower) while mesenteric depot was not affected at all. CLA1 group experienced only a significant reduction in retroperitoneal fat depot.

**Table 2:** Adipose tissue weights in mice supplemented with CLA

	Control	CLA1	CLA2
**White adipose tissues**			
Epididymal (g)	0.644 ± 0.048^a^	0.628 ± 0.057^a^	0.284 ± 0.022^b^
Retroperitoneal (g)	0.212 ± 0.030^a^	0.129 ± 0.015^b^	0.069 ± 0.007^c^
Mesenteric (g)	0.250 ± 0.020	0.262 ± 0.022	0.233 ± 0.018
*Sum *(g)	1.107 ± 0.089^a^	1.018 ± 0.091^a^	0.586 ± 0.042^b^
**Brown adipose tissue **(g)	0.118 ± 0.007^a^	0.126 ± 0.007^a^	0.095 ± 0.003^b^

Weights of white adipose tissues from different anatomical locations and brown adipose tissue of mice treated with a daily dose of CLA equivalent to 3 mg CLA/animal in CLA1 group and 10 mg/animal in CLA2 group for the first 30 d and 6 mg CLA/animal in CLA1 group and 20 mg/animal in CLA2 group for the subsequent 35 d of treatment. Data are expressed in grams and are the means ± SEM of 8 mice. Means in a row without a common letter differ, *P *< 0.05 (ANOVA followed by LSD test).

### Liver weight and triglyceride content

There was no effect of CLA treatment on the weight of liver (0.876 ± 0.03 in control, 0.932 ± in CLA1 and 0.970 ± 0.052 g in CLA2 group, n = 8). No changes in hepatic triglyceride content were observed after CLA treatment (50.49 ± 2.98 in control, 55.66 ± 2.12 in CLA1 and 50.72 ± 3.36 mg triglycerides/g liver, n = 8).

### Plasma parameters

Plasma glucose, adiponectin and leptin concentrations were not different between groups after 30 days of treatment (Table 3).

**Table 3:** Effects of CLA treatment on plasma concentration of metabolites in mice

	Control	CLA1	CLA2
*30 days of treatment*			
Glucose (mmol/L)	8.2 ± 0.2	7.6 ± 0.2	8.0 ± 0.3
Adiponectin (μg/ml)	13.41 ± 1.35	13.78 ± 0.67	13.64 ± 1.14
Leptin (ng/ml)	3.11 ± 0.88	2.08 ± 0.35	1.62 ± 0.27
*65 days of treatment*			
Glucose (mmol/L)	4.33 ± 0.23^ab^	4.15 ± 0.14^a^	4.90 ± 0.21^b^
Adiponectin (μg/ml)	17.33 ± 1.05^a^	16.87 ± 0.84^a^	11.64 ± 1.25^b^
Leptin (ng/ml)	2.09 ± 0.39^a^	2.57 ± 0.45^a^	0.45 ± 0.10^b^
Resistin (ng/ml)	15.27 ± 1.04	15.90 ± 0.91	14.32 ± 0.93
NEFAs (mg/dl)	26.00 ± 1.80^a^	19.36 ± 1.96^b^	14.44 ± 1.47^b^
Glycerol (mg/ml)	0.19 ± 0.02^a^	0.15 ± 0.02^a^	0.06 ± 0.02^b^
Triglycerides (mg/ml)	0.61 ± 0.03	0.60 ± 0.05	0.51 ± 0.08
Insulin (pmol/L)	15.95 ± 0.35^a^	18.50 ± 0.87^b^	19.89 ± 0.80^b^
Leptin/adiponectin ratio	0.12 ± 0.02^a^	0.16 ± 0.03^a^	0.04 ± 0.01^b^
HOMA-IR	0.42 ± 0.02^a^	0.47 ± 0.02^a^	0.60 ± 0.04^b^
R-QUICKI	0.46 ± 0.01	0.48 ± 0.01	0.49 ± 0.01

At 30 d of treatment and after 3 h fast, glucose, adiponectin and leptin plasma concentrations were determined from tail blood samples. The rest of plasmatic metabolites were determined at the end of the study (65 days of treatment) after 10 h fast and from blood samples collected by cardiac puncture. Data are means ± SEM of 8 mice at 65 d of treatment; of 3-8 mice for leptin and adiponectin on day 30 and of 7 mice for glucose on day 30. Means in a row without a common letter differ, *P *< 0.05 (ANOVA followed by LSD test).

Adiponectin and leptin concentrations were significantly decreased with the highest dose of CLA at the end of the study (Table 3). No significant differences in circulating resistin concentration were found between groups and the same was seen concerning plasma triglycerides. NEFA concentration decreased in both CLA treated groups while plasma glycerol concentration decreased only in CLA2 group (Table 3). Insulin concentration increased with CLA treatment (*P *< 0.01) (16% and 25% in CLA1 and CLA2 group, respectively) and CLA2 group presented higher fasting glucose concentration than CLA1 (*P *< 0.05) (Table 3).

### ITT and calculated indices

No differences between groups were observed in the ITT carried out at day 30 of treatment, either measuring the change in plasma glucose concentration (data not shown) or the area under the curve (756 ± 41 in control, 794 ± 46 in CLA1 and 913 ± 81 mmol glucose·min/L in CLA2 group, n = 5-6). The calculated homeostatic model assessment for insulin resistance (HOMA-IR) was higher in CLA2 group at the end of the study (Table 3) (*P *< 0.001). However, the calculated revised quantitative insulin sensitivity check index (R-QUICKI) showed no differences between groups.

### Gene expression in adipocytes and SVF

Adiponectin and leptin mRNAs were dose-dependently reduced by CLA treatment in mature adipocytes (*P *< 0.001). MCP1 mRNA expression was reduced with the highest dose with respect to both control and CLA1 groups, while it was increased in adipocytes from CLA1 with respect to both control and CLA2 groups (Table 4).

**Table 4:** Relative expression of target mRNAs in mature adipocytes and stromal vascular fraction in mice treated with CLA

	Control	CLA1	CLA2
**Mature adipocytes**			
Adiponectin	1.00 ± 0.05^a^	0.72 ± 0.04^b^	0.39 ± 0.02^c^
Leptin	1.00 ± 0.07^a^	0.57 ± 0.05^b^	0.16 ± 0.01^c^
MCP1	1.00 ± 0.09^a^	1.28 ± 0.10^b^	0.71 ± 0.06^c^
**Stromal Vascular Fraction**			
IL-6	1.00 ± 0.09^a^	0.96 ± 0.10^a^	0.61 ± 0.04^b^
TNFα	1.00 ± 0.11^a^	1.47 ± 0.18^ab^	1.87 ± 0.30^b^
iNOS	1.00 ± 0.08	0.81 ± 0.05	1.24 ± 0.20
Emr1	1.00 ± 0.11^a^	1.46 ± 0.11^a^	2.64 ± 0.28^b^
MCP1	1.00 ± 0.13	1.40 ± 0.18	1.23 ± 0.11

Epididymal adipose tissue was digested by collagenase and then separated into mature adipocytes and stromal vascular fraction. Expression levels of target genes of each fraction were measured by real time PCR and normalized by the internal housekeeping gene 1*8S *rRNA. The results, mean ± SEM of 6-8 mice/group, are expressed as fold induction over control group. Means in a row without a common letter differ, *P *< 0.05 (ANOVA followed by LSD test).

No effects of CLA treatment on iNOS and MCP1 gene expression were appreciated on the SVF (Table 4). The highest CLA dose achieved a reduction in IL-6 gene expression (*P *< 0.05) and an increase in Emr1 (*P *< 0.001) with respect to both control and CLA1 groups. Meanwhile TNFα gene expression was increased in SVF of CLA2 animals with respect to the control group (*P *< 0.01) (Table 4).

**Figure 2:**
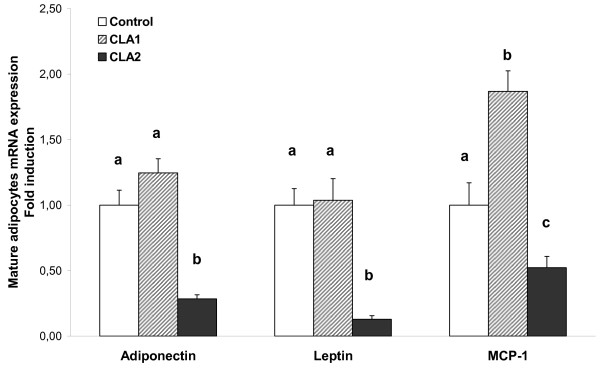
**Contribution of mature adipocytes isolated from epididymal fat depot to the expression of target mRNA in CLA treated mice**. Epididymal adipose tissue was digested by collagenase and then separated into mature adipocytes and stromal vascular fraction. Expression levels of target genes of each fraction were measured by real time PCR and normalized by the internal housekeeping gene *18S *rRNA. Expression data in adipocytes, derived from equal amount of RNA (Table 4), were referred to the total RNA content in the adipocyte fraction. Data, means ± SEM of 7-8 mice, are represented as fold induction over control group. Mean values with unlike letters are significantly different (*P *< 0.01); ANOVA followed by LSD test.

**Figure 3:**
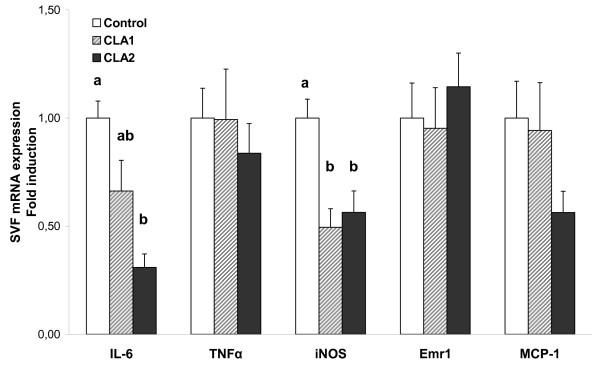
**Contribution of SVF cells isolated from epididymal fat depot to the expression of target mRNA in CLA mice**. Epididymal adipose tissue was digested by collagenase and then separated into mature adipocytes and stromal vascular fraction. Expression levels of target genes of each fraction were measured by real time PCR and normalized by the internal housekeeping gene *18S *rRNA. Expression data in the stromal vascular fraction (SVF), derived from equal amount of RNA (Table 4) were referred to the total RNA content in SVF. Data, means ± SEM of 6-8 mice, are represented as fold induction over control group. Mean values with unlike letters are significantly different (P < 0.01); ANOVA followed by LSD test.

Interestingly, CLA treatment showed a tendency to increase RNA yield, particularly in the adipocyte fraction where it attained statistical significance (Table 5). This is of special relevance because of the minor size of adipose depots in mice treated with CLA. For this reason, gene expression in mature adipocytes (Figure 2) and SVF (Figure 3) was referred to the total RNA content of the respective epididymal fraction in order to attain a closer physiological view of the endocrine function of the fat depot and its potential for macrophage recruitment. Under this novel perspective, gene expression data showed a slightly different profile than above, whereas MCP1 adipose gene expression was unaffected, adiponectin and leptin decreased only with the highest dose of CLA in mature adipocytes (Figure 2), therefore, total contribution of mature adipocytes reflected in a better way, the plasma levels of the two adipocytokines. Concerning the expression profile in cells from the SVF, the decrease in IL-6 gene expression with the highest dose was maintained (Figure 3) and the same tendency was now evident in CLA1 group. Furthermore, TNFα and Emr1 gene expression were not affected by CLA treatment, while iNOS gene expression decreased with both doses of CLA. A reduction of MCP1 gene expression, although not statistically significant, was observed with CLA2 dose in relation to control group.

**Table 5:** Total RNA yields obtained from mature adipocytes and stromal vascular fraction in CLA treated mice

	RNA yield (μg RNA/g of epididymal depot)		
	**Control**	**CLA1**	**CLA2**
**Mature adipocytes**	5.5 ± 0.6^a^	9.8 ± 0.7^b^	8.8 ± 0.8^b^
**Stromal vascular fraction**	8.8 ± 0.4	6.1 ± 1.2	11.0 ± 2.5

Epididymal adipose tissue was digested by collagenase and then separated into mature adipocytes and stromal vascular fraction. RNA extracted from each fraction was quantified and referred per gram of epididymal adipose tissue weight. Data are expressed in μg RNA per g of epididymal tissue and are the means ± SEM of 7-8 mice/group. Means in a row without a common letter differ, *P *< 0.05 (ANOVA followed by LSD test).

## Discussion

Mice constitute an animal model particularly sensitive to potential deleterious side effects of CLA such as insulin resistance, hyperinsulinaemia and liver steatosis [7-11]. Most previous studies have made use of enriched diets, containing between 0.5 and 1.5% CLA (for review, see [6,19]) which in this animal model, supply a daily dose around 50 times higher than those successfully used in human trials [12,20]. Therefore, the adverse side effects seen in mice could be due to the use of large doses rather than the use of optimal doses which would reduce body fat content without showing any of the adverse effects reported. According to this hypothesis, we have previously reported that mice fed with a standard-fat diet and treated with moderate doses of the commercial product Tonalin^® ^- an equimolar mix of *cis*-9, *trans*-11 and *trans*-10, *cis*-12 CLA isomers - show reduced body weight gain and lower fat depots without any of the adverse effects associated with CLA treatments [16]. Therefore, similar doses of the CLA mixture were used in the current study, in order to assess their efficacy in animals with susceptibility to diet-induced body weight gain and exposure to a high-fat diet.

A slight effect on reducing body weight gain with CLA treatment was observed by the end of the study. In fact, the lower dose of CLA reduced only the retroperitoneal fat depot, which seems to be the most sensitive to CLA effects [16,21,22], whereas the highest dose also reduced the size of the epididymal depot, supporting the fact that CLA effects are tissue-specific as seen in humans [23]. In brief, the administration of CLA reduces the gain of weight and fat observed in control group, suggesting that the administration of CLA may mitigate the effects of an obesity-promoting environment.

A reduction in adiposity is usually associated with improved insulin sensitivity and plasma adipocytokine profile, but this is not so clear when the fat loss is caused by CLA supplementation. While some studies have shown beneficial effects in rat models [24-26] and in mice [16,25], several have observed harmful effects of CLA on insulin sensitivity, particularly in mice [8,9,11,14,27]. Here, the higher dose of CLA caused a reduction in both, plasma leptin and adiponectin concentrations (Table 3); which could be attributed to the reduction of fat depots, the main synthesizing organs, and also to the reduction in absolute terms of its gene expression (Figure 2). Deregulation in the production of these two adipocytokines has been observed in both obese and lipodystrophy states [28] and has been proposed to contribute to the impairment of insulin sensitivity [8]. Lipodystrophy may occur in mice treated with high doses of CLA, due to its higher sensitivity to the CLA-induced reduction in body fat [11,29,30]. In these conditions, the drastic plasma reduction of leptin and adiponectin associated with CLA treatment, induces fatty liver and hyperinsulinaemia, not through the direct induction of hepatic lipid synthesis and insulin resistance, but because of the scarcity of the adipose tissue [30]. Interestingly, the doses of CLA tested here were associated with an important reduction in body fat, but without reaching the lipodystrophy status. Although fat loss was accompanied by a moderate degree of hyperinsulinaemia (25% increase) it was far from the 300-400% increase found in other studies using higher doses [8,10,31]. No hepatic steatosis or liver enlargement was observed and it was accompanied by maintenance of insulin sensitivity, as particularly indicated by ITT and R-QUICKI, despite the higher HOMA-IR index. In fact, R-QUICKI has been described as more accurate than HOMA-IR, as surrogate marker to assess insulin sensitivity incorporating the level of fasting NEFA together with insulin and glucose levels [32,33]. In consequence, the decreased circulating concentrations of leptin and adiponectin promoted by CLA treatment were consistent with maintenance of glucose-insulin homeostasis, as seen in normal-fat fed mice, where CLA causes fat loss, decreases leptin and goes in hand with lower adiponectin levels, reaching a novel set point between these two circulating adipocytokines, which is associated with the maintenance of insulin sensitivity and a decrease in the expression of inflammatory markers in adipose tissue [16]. The relative amount of these two adipocytokines is likely to be more important than their absolute concentrations. Thus, for example, lipoatrophy-associated insulin resistance can be completely reversed by the combination of adiponectin and leptin, but only partially by either adiponectin or leptin alone [28].

Concerning the effects of CLA on the inflammatory profile of adipose tissue, supplementation with CLA may induce inflammatory gene expression in adipocytes and promote macrophage infiltration into adipose tissue showing isomer specific dependence as seen for *trans*-10, *cis*-12 CLA [13,14,34,35] but not for *cis*-9, *tras*-11 CLA [15] either for the mix of both isomers under normal fat diet [16].

In accordance with the minor outcome on fat reduction, minor effects on gene expression were also seen in the group that received the lowest dose of CLA, whereas the highest dose of CLA had a major impact on adipose and SVF gene expression profile. Expression of MCP1, a chemoattractant protein which promotes recruitment of macrophages into adipose tissue and, therefore, inflammatory responses in obesity [36], was decreased in adipocytes (Figure 2) and showed the same tendency in SVF of CLA2 group (Figure 3). This was accompanied by a reduction in the expression of pro-inflammatory mediators such as IL-6 and iNOS and unaltered expression of both TNFα and the macrophage marker Emr1. Proinflammatory cytokines have been shown to promote adipocyte delipidation and impair insulin signaling [13,37,38]. In fact, *trans*-10, *cis*-12 CLA was reported to induce IL-6 secretion which seemed to be, at least in part, responsible for the isomer-mediated suppression of PPARγ target gene expression and impairment of insulin sensitivity in mature human adipocytes [13]. Collectively, our data suggested that, particularly at the highest dose tested, CLA supplementation may ameliorate the inflammatory state in obesity, attenuating macrophage infiltration and/or activation into adipose tissue, as seen in animals fed with a standard-fat diet [16] but not with higher doses [10] or by administration of the single *trans*-10, *cis*-12 CLA isomer [14].

## Conclusion

In conclusion, an equimolar mix of the two main CLA isomers, at a moderate dose, was able to mitigate body fat accumulation by high fat feeding, and in contrast to studies with larger doses of CLA and particularly with pure *trans*-10, *cis*-12 isomer, this was associated with an improvement of the lipid profile in plasma and maintenance of insulin sensitivity, despite a moderate degree of hyperinsulinaemia, which was far from the 3-4 fold increase observed with higher doses and *trans*-10, *cis*-12 isomer. Furthermore, in our experimental conditions, CLA seems to ameliorate the inflammatory profile in adipose tissue, causing a reduction in the expression of MCP1, the main macrophage recruitment factor, and a decrease in the expression of the pro-inflammatory mediators iNOS and IL-6.

## Competing interests

The authors declare that they have no competing interests.

## Authors' contributions

PP was responsible for animal care, experimental work, acquisition of data, statistical analysis, and manuscript preparation. She has also collaborated in study design and interpretation of data. FS and AP have equally contributed to the conception and design of the study, interpretation of data and drafting of the manuscript.

All authors read and approved the final manuscript.
